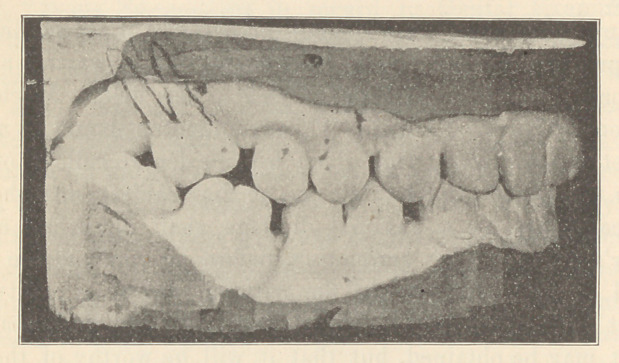# Pains from Malocclusion

**Published:** 1899-12

**Authors:** John S. Engs

**Affiliations:** Oakland, Cal.


### PAINS FROM MALOCCLUSION.

BY JOHN S. ENGS, D.D.S., OAKLAND, CAL.

In the fall of 1897 Mr. F., aged about forty years, had occasion to visit me for treatment. Among other things, I filled a distal cavity in the right superior twelfth-year molar with Flagg's contour alloy.

Three months later the patient returned complaining of slight pain in that tooth. As the cavity had been quite deep, I suspected pulpitis, and made an application to the gum over the tooth, using tincture of iodine, tincture of aconite, and chloroform P. E. The patient was seen the next day; he said all was well.

Six weeks later he called again, with recurrence of the trouble. The same treatment was repeated, but the next day the pain returned. I repeatedly touched the gums for several days, but the relief from pain was only temporary. The patient suffered more than before, especially while chewing food and when any pressure was brought to bear on this tooth. With the exception of decided evidence of vitality in the pulp, I had every reason to think it a pronounced case of pericementitis threatening an abscess. I was not quite satisfied with the diagnosis, and, suspecting trouble from the position of the tooth, took impressions of both jaws, that I might look closer into the articulation.

The accompanying illustration shows fairly well the relative positions of the teeth. The superior twelfth-year molar had been thrown so far forward from its normal position that it almost touched the second bicuspid (both the superior and inferior sixth-year molars had long since been extracted).

Every closure of the lower jaw brought the coronal surface of the inferior wisdom-tooth to bear distally upon the upper second molars, causing a severe lateral strain.

I ground away the corono-distal surface of the upper second molar, and then forced a wedge of wood between it and the second bicuspid. The patient could then chew without any discomfort. It was evident that the trouble was due to malocclusion.

It seemed proper that the upper second molar should be moved back as nearly as possible to its normal position. This was effected with the aid of cottonwood wedges inserted between it and the second bicuspid. The process was begun May 2, and completed

June 8, 1898. A gold band with a nugget of gold soldered to it, large enough to fill the space between the two teeth, was cemented to the molar. More than a year has elapsed since then and there has been no recurrence of the trouble.

The lines on the plaster cast, showing approximately the position of the molar roots, are drawn on the cast in pencil, and are not reproductions of the true roots.

## Figures and Tables

**Figure f1:**